# Indoleamine 2, 3-Dioxygenase-Mediated Tryptophan Catabolism: A Leading Star or Supporting Act in the Tuberculosis and HIV Pas-de-Deux?

**DOI:** 10.3389/fcimb.2019.00372

**Published:** 2019-10-29

**Authors:** Clement Gascua Adu-Gyamfi, Dana Savulescu, Jaya Anna George, Melinda Shelley Suchard

**Affiliations:** ^1^Centre for Vaccines and Immunology, National Institute for Communicable Diseases, Johannesburg, South Africa; ^2^Department of Chemical Pathology, Faculty of Health Sciences, School of Pathology, University of the Witwatersrand, Johannesburg, South Africa

**Keywords:** IDO, latent TB, active TB, kynurenine/tryptophan ratio, nicotinamide

## Abstract

Progression from latency to active Tuberculosis (TB) disease is mediated by incompletely understood host immune factors. The definitive characteristic of progressive human immunodeficiency virus (HIV) disease is a severe loss in number and function of T lymphocytes. Among the many possible mediators of T lymphocyte loss and ineffective function is the activity of the immune-modulatory enzyme indoleamine 2,3-dioxygenase (IDO). IDO is the rate-limiting enzyme converting tryptophan to kynurenine. IDO activity was initially recognized to mediate tolerance at the foeto-maternal interface. Recently, IDO activity has also been noted to play a critical role in immune tolerance to pathogens. Studies of host immune and metabolic mediators have found IDO activity significantly elevated in HIV and TB disease. In this review, we explore the link between IDO-mediated tryptophan catabolism and the presence of active TB disease in HIV-infected patients. We draw attention to increased IDO activity as a key factor marking the progression from latent to active TB disease in HIV-infected patients.

## Introduction

Recently, the effect of indoleamine 2,3-dioxygenase-1 (IDO) activity in various diseases, including tuberculosis (TB) and human immunodeficiency virus (HIV) disease has generated considerable interest. Tryptophan, an essential amino acid in the body, can either be converted to serotonin or oxidized to kynurenines. IDO is the rate-limiting enzyme in the conversion of tryptophan to kynurenines (Higuchi and Hayaishi, 1967; Katz et al., 2008). IDO activation plays a crucial role in macrophage and dendritic cell modulation toward alternate activation and immune tolerance (Nagamatsu and Schust, 2010; Martinez and Gordon, 2014). Two theories have been postulated to explain the mechanism of tolerance induced by IDO. Firstly, depletion of tryptophan in the microenvironment starves T cells of a vital micronutrients, which results in the inability of T cells to proliferate and function effectively (Fallarino et al., 2006; Sharma et al., 2009; Yan et al., 2010). Secondly, the accumulation of kynurenine and its downstream metabolites promotes T cell stress, anergy and apoptosis (Frumento et al., 2002; Fallarino et al., 2006; Mezrich et al., 2010; Platten et al., 2012). IDO activity plays a crucial role in T cell hypo-responsiveness during pregnancy and evasion of malignant cells from immune surveillance (Munn et al., 1998; Mellor and Munn, 1999, 2004; Nagamatsu and Schust, 2010; Platten et al., 2012). IDO is expressed in many malignancies and high IDO expression is associated with poor prognosis in a variety of cancer types (Brandacher et al., 2006; Ino et al., 2006; Creelan et al., 2013; Wei et al., 2018). Prior to 1998, IDO activity was considered to be anti-microbial, acting via depletion of available tryptophan, which is required for microbial growth (Däubener and Mackenzie, 1999). IDO activity, however, may have opposing roles, either by dominantly suppressing pathogen replication or providing a comfortable immunological niche for the pathogen to thrive in and to evade immune-mediated killing (Leonhardt et al., 2007; Almeida et al., 2009; Divanovic et al., 2011; Makala et al., 2011; Yeung et al., 2012). Both HIV-infection and TB disease are associated with upregulation of IDO activity (Divanovic et al., 2011; Blumenthal et al., 2012; Chen et al., 2014). In this review, we highlight evidence examining the role of elevated IDO activity in HIV infection and active TB disease.

## Tryptophan and Its Cellular Metabolism

Essential micronutrients are those which cannot be produced endogenously and must be acquired through dietary sources. Humans, like other mammals, cannot synthesize tryptophan, and therefore, must obtain it from their diet and internal protein turn-over (Brown, 1996). Foods such as banana, spinach, soya-beans, fish, egg, milk, and cheese are rich sources of tryptophan (Sainio et al., 1996; Friedman, 2018). Human beings require about 150–250 mg tryptophan from the diet, and this amount is exceeded in regular household meals (Peters, 1991). Dietary tryptophan is absorbed and delivered through the hepatic portal system, where it enters the bloodstream for protein synthesis and other cellular functions.

In certain cell types, tryptophan can be converted to serotonin or melatonin by tryptophan hydroxylase (Ruddick et al., 2006). Alternatively, tryptophan can be catabolised to kynurenines (Higuchi and Hayaishi, 1967; Takikawa, 2005; Katz et al., 2008). Two cytosolic enzymes, IDO and Tryptophan 2,3-dioxygenase (TDO), are responsible for tryptophan catabolism in specific cell types (Takikawa, 2005). IDO and TDO are intracellular, non-secreted enzymes (Yamazaki et al., 1985). While TDO expression and activity is confined to hepatocytes, IDO is expressed by a wider variety of cells, but most potently by macrophages, dendritic and mesenchymal stem cells (Thomas et al., 2001; Dai and Zhu, 2010; Francois et al., 2010). IDO comprises two related, but distinct enzymes encoded by two different genes, namely *IDO1* and *IDO2* (Yamazaki et al., 1985; Takikawa, 2005; Bilir and Sarisozen, 2017). Both enzymes are expressed by epithelial and antigen-presenting cells (Munn et al., 1998; Nagamatsu and Schust, 2010; Zhu, 2010). In this review, IDO1 is referred to as IDO. IDO or TDO initiate catabolism of tryptophan by oxidizing the indole ring to produce N-formylkynurenine (Liu et al., 2016). N-formylkynurenine can be further converted to stable end-products including kynurenic acid, anthranilic acid and 3-hydroxykynurenine. N-formylkynurenine can also be further oxidized to nicotinamide adenine dinucleotide (NAD+), a vital co-factor in energy production, DNA synthesis and cellular homeostasis (Bryleva and Brundin, 2017). The tryptophan-kynurenine-NAD+ pathway represents the *de novo* pathway for NAD+ synthesis when niacin intake is limited in the diet (Higuchi and Hayaishi, 1967; Takikawa, 2005).

## IDO Expression and Function

Munn and colleagues observed IDO expression and activity at the foeto-maternal interface in pregnant mice (Munn et al., 1998). Their findings suggested that IDO activity mediated immune tolerance to the fetus by the mother. Interestingly, blocking IDO activity in allogeneic pregnancy led to a break in the tolerogenic state that protected the fetus from the maternal immune system (Munn et al., 1998; Mellor and Munn, 1999). Since then, several studies have shown that IDO is expressed in various tissues including liver, lymph nodes, spleen and tonsils (Mellor and Munn, 2004; Bilir and Sarisozen, 2017). Antigen-presenting cells, particularly macrophages and dendritic cells, can express IDO in response to cytokines such as IFN-γ and TNF-α (Robinson et al., 2005; Nagamatsu and Schust, 2010). During infection or inflammation, IDO is stimulated in polymorphonuclear cells, endothelial cells, epithelial cells, eosinophils and fibroblasts by pro-inflammatory cytokines (Hayashi et al., 2004; Robinson et al., 2005; Xu et al., 2008).

## IDO-mediated Tryptophan Metabolism and Host Immunity

Tryptophan depletion and subsequent generation of kynurenine metabolites have considerable effects on the host's immunity. The IDO pathway plays a significant role in T cell hypofunctionality, a critical concept in immune tolerance to pathogens (Mellor et al., 2017). In acute inflammation, induction of IDO may act as a regulatory feedback loop to prevent over-reaction of the immune response (Grohmann et al., 2003). In chronic inflammatory conditions, elevated IDO activity may dampen the host's protective immunity (Leonhardt et al., 2007; Divanovic et al., 2011).

**Figure 1:**
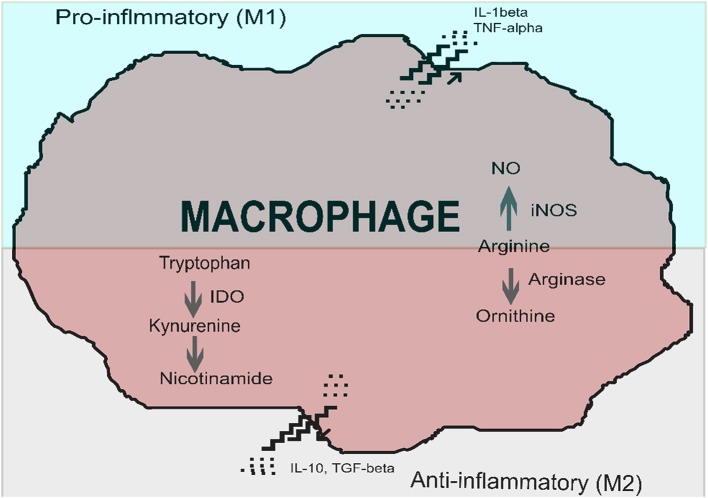
Macrophage activation, in the presence of indoleamine 2,3-dioxygenase (IDO), favors polarization to an immunosuppressive phenotype (alternatively activated macrophage) over that of a pro-inflammatory macrophage (classically activated macrophage). Classically activated macrophages produce pro-inflammatory cytokines such as interleukin 1-β (IL-1β) at the time of macrophage activation. Alternatively activated macrophages produce immune-suppressive cytokines such as interleukin 10 (IL-10) and transforming growth factor-beta (TGF-β). Key enzymes involved in classical activation include inducible nitric oxide synthase (iNOS), which converts arginine into nitric oxide. Two critical enzymes are involved in the alternative activation pathway: arginase converts arginine to ornithine, and indoleamine 2,3-dioxygenase converts tryptophan to kynurenines. Image adapted and modified from Nagamatsu and Schust (2010) with permission.

Immune cells, in particular macrophages and dendritic cells, are sites of IDO production. IDO expression and activation creates a local immunosuppressive micro-environment, which allows pathogens to escape sterilizing immunity or containment (Nagamatsu and Schust, 2010). IDO induction is a feature of alternative macrophage activation, which favors a tolerogenic or anti-inflammatory state, rather than a pro-inflammatory phenotype. While a pro-inflammatory phenotype leads to killing and elimination of a pathogen, an alternatively activated phenotype leads to immune tolerance, induction of regulatory T cells, tissue repair and fibrosis (Nagamatsu and Schust, 2010; Mellor et al., 2017). Macrophage phenotypes have been termed M1 and M2, however, plasticity and intermediate types exist (Das et al., 2015). The alternative pathway of macrophage activation is shown in Figure 1.

Downstream metabolites of the IDO pathway such as quinolinic acid, are neurotoxic and have been implicated in HIV-associated dementia (Gostner et al., 2015). Increased tryptophan oxidation leads to a net increase in circulating niacin, which increases the basal metabolic rate (Murray, 2003a). Kynurenines also activate the aryl hydrocarbon receptor (AhR), a transcription factor, key in sensing external environmental triggers and modulating gene expression in response (Lewis et al., 2014; Schmidt and Schultze, 2014; Salazar et al., 2017). Lipopolysaccharides, viral proteins such as HIV-Tat, HIV-Nef, and HIV-p17, and pathogen-associated molecular patterns, stimulate IDO expression to influence survival and pathogenesis (Murray, 2003b, Schmidt and Schultze, 2014). In malignancies, where IDO activity has been extensively studied, IDO activity correlates with dampened immunity, and poor prognosis (Ino et al., 2006; Hornyák et al., 2018).

## Tryptophan Metabolism in Microbial Infections

Amino acids are critical micronutrients for all pathogens. Microbes either synthesize amino acids or depend on their hosts for amino acids for growing and multiplying. The ability of the host to deprive pathogens of essential micronutrients is a crucial feature of innate defense. Tryptophan, just like carbon, iron, and nitrogen are critical for microbial survival (Rohmer et al., 2011). Conventionally, tryptophan depletion has long been known to have anti-microbial actions for pathogens, which cannot synthesize it from smaller molecules (Russell, 2013; Zhang and Rubin, 2013; Zhang et al., 2013). Tryptophan deprivation has been shown to inhibit Chlamydia and Toxoplasmosis *in vitro* (Byrne et al., 1986; Murray et al., 1989). Several *in vitro* studies have reported that measles, influenza, cytomegalovirus, and herpes simplex viral infections are also susceptible to tryptophan depletion (Obojes et al., 2005; Zhang and Rubin, 2013; Schmidt and Schultze, 2014). On the other hand, mycobacteria possess all the complete set of tools to synthesize any essential amino acid, including tryptophan, required for their survival, a feature which gives them an evolutionary advantage (Zhang and Rubin, 2013; Zhang et al., 2013).

## Animal Studies of IDO Activity in TB Pathogenesis

In animal models, increased tryptophan metabolism via the IDO pathway has been associated with poor immune responses to M. TB (O'Connor et al., 2009; Mehra et al., 2012; Zhang and Rubin, 2013; Zhang et al., 2013). IDO activation has been reported in mycobacterial infection in both mice and non-human primates (Mehra et al., 2012; Foreman et al., 2016; Gautam et al., 2018). In mice, increased IDO activation has been associated with poor TB outcomes (Moreau et al., 2005; O'Connor et al., 2009). In macaques, Mehra and colleagues demonstrated that IDO induction in the periphery of the granuloma correlates with active TB disease (Mehra et al., 2012). IDO induction was significantly higher in mock-vaccinated compared with BCG-vaccinated animals, and was seen predominantly in non-lymphocytic cells within the ring structure of the granuloma, lining the area of central necrosis (Mehra et al., 2012). Gautam et al. (2018) also showed that M. TB induces IDO expression and activity in the lung macrophages of macaques with active TB disease (Mehra et al., 2012). Based on evidence from both human and animal studies, M. TB infection is associated with high expression of IDO in the granuloma, leading to compromised T cell function, which promotes mycobacterial survival and growth (Mehra et al., 2012; Seo et al., 2015; Gautam et al., 2018). Blocking IDO expression and activity led to reduced pathogen burden, minimal TB pathology and mproved survival (Gautam et al., 2018). Furthermore, IDO inhibition was accompanied by restoration of T cell quantity and functions (Gautam et al., 2018). Collectively, these findings indicate that IDO activation plays a crucial role in M. TB pathogenesis in animal models.

## Tryptophan Catabolism in HIV Infection

When co-infecting a host, HIV and M. TB influence each other, accelerating deterioration of immune function. HIV infection causes severe loss of T cells, both in number and function (Douek et al., 2001; Alimonti et al., 2003; Schindler et al., 2006). Specific mechanisms of immune impairment are beyond the scope of this review; however, it is noted that aspects of T cell exhaustion persist in the face of chronic immune activation (Brenchley et al., 2006; Doitsh et al., 2014; Doitsh and Greene, 2016; Sokoya et al., 2017). In the last three decades, a wealth of information has indicated that IDO activation and its associated metabolites play a role in human HIV infection and early progression to AIDS (Fuchs et al., 1990; Hunt et al., 2014; Jenabian et al., 2015; Mehraj and Routy, 2015; Gelpi et al., 2017). Tryptophan catabolism and its immune-regulatory effect are notable during HIV progression (Almeida et al., 2009; Mehraj and Routy, 2015; Gelpi et al., 2017; Gautam et al., 2018). There is a strong association of elevated IDO activity with AIDS-defining illnesses (Nagamatsu and Schust, 2010; Catalfamo et al., 2012; Martinez and Gordon, 2014; Sokoya et al., 2017). Furthermore, gut microbial translocation sustains increased immune activation, and chronic immune activation results in a pro-inflammatory cytokine storm, which activates the IDO pathway (Vazquez-Castellanos et al., 2015; Chen et al., 2018; Lu et al., 2018).

Werner et al. (1988) reported that HIV-infected patients have high serum kynurenine-to-tryptophan ratio (IDO activity). The elevated kynurenine-tryptophan ratio in HIV-infected patients correlates with advanced HIV disease (Huengsberg et al., 1998). Huengsberg et al. (1998) observed a striking association between the kynurenine-tryptophan ratio, declining CD4 T cells and progression to AIDS. Antiretroviral therapy (ART) improved the kynurenine-tryptophan ratio and correlated with decreased viral load and improved CD4 cell count (Zangerle et al., 2002). Hunt et al. (2014) showed that the kynurenine-tryptophan ratio was an independent predictor of death. Multiple studies have reported a similar finding of increased IDO activity or kynurenine metabolites in HIV infection (Table 1). We speculate that increased IDO-mediated tryptophan catabolism in HIV infection may be more than merely an association, but play a causal role in susceptibility to disease, particularly active TB.

**Table 1:** Studies reporting elevated IDO activity, increased kynurenine or decreased tryptophan in human HIV infection.

**References**	**Participant demographics**	**ART therapy**	**Sample type**	**Method of IDO** **quantification**	**Major conclusion (s)**
Werner et al. (1988)	HIV+ patients (*n* = 11) HIV– patients (*n* = 11)	Not specified	Serum	HPLC-MS	Elevated kynurenine/tryptophan ratio suggested increased tryptophan catabolism in HIV-infected patients. Increase tryptophan metabolism may have contributed to neurologic symptoms found among HIV-infected patients.
Larsson et al. (1989)	HIV+ patients (*n* = 24) HIV– patients (*n* = 14)	No	Blood and CSF	HPLC-MS	HIV patients had significantly decreased tryptophan in blood and CSF compared to controls. HIV patients with severe AIDS-defining symptoms such as markedly lower CD4 count had significantly lower tryptophan concentrations.
Fuchs et al. (1990)	HIV+ patients (*n* = 22) HIV– patients (*n* = 14)	Not specified	Serum and CSF	HPLC-MS	Decreased tryptophan levels were associated with chronic immune activation in HIV positive patients. IDO activity induced by interferon-γ may be the cause of reduced tryptophan concentration in HIV infected patients.
Wiegand et al. (1991)	HIV+ patients (*n* = 14) HIV– patients (*n* = 14)	95% on Zidovudine monotherapy	Serum and CSF	HPLC-MS	Lower tryptophan concentration in HIV-infected patients was associated with sleep disturbances.
Fuchs et al. (1991)	HIV+ patients (*n* = 42) Controls were HIV– from three different cohorts	38% on only Zidovudine	serum	HPLC-MS	Decreased tryptophan concentration in HIV-infected patients may be due to chronic immune activation which result in increased IDO activation.
Heyes et al. (1991, 1992)	HIV+ patients (*n* = 126) Controls HIV– (*n* = 28)	No	Serum and CSF	HPLC-MS	Tryptophan concentration decreased with increased kynurenines in serum and cerebrospinal fluid.
Hortin et al. (1994)	HIV+ patients (*n* = 20) HIV– patients (*n* = 20)	Majority on ARV	Plasma	Flow cytometry and ELISA	Plasma tryptophan concentration decreases in response to HIV infection.
Gisslén et al. (1994)	HIV+ patients (n = 14)	Yes	Blood and CSF	HPLC-MS	Initiation of antiretroviral therapy was associated with decreased immune activation and decreased tryptophan breakdown in HIV-infected patients.
Huengsberg et al. (1998)	HIV+ patients (*n* = 206) HIV– patients (*n* = 72)	Not specified	Serum	HPLC-MS	Kynurenine/tryptophan ratio showed reciprocal relation to tryptophan. Severe HIV disease correlated with increased tryptophan degradation and increased IDO activity.
Look et al. (2000)	HIV+ patients (*n* = 17) HIV– patients (*n* = 55	Yes	Serum	GC-MS	HIV-infected patients had increased kynurenine/tryptophan ratio compared to controls. ART treatment mitigated immune activation in HIV disease, and led to a decrease in IDO activation.
Murray et al. (2001)	HIV+ patients (*n* = 4)	Yes	Plasma	HPLC-MS	Plasma tryptophan increased 40% in HIV-infected patients following treatment with antiretroviral medications, however, other amino acids concentrations remained fairly unchanged in plasma.
Zangerle et al. (2002)	HIV+ patients (*n* = 45) HIV– patients (*n* = 40)	Yes	Plasma	HPLC-MS	Tryptophan degradation was increased in HIV-infection, and ART partially reversed it.
Atlas et al. (2007)	HIV+ patients (*n* = 22) HIV– patients (*n* = 22)	Yes	CSF	HPLC-MS	HIV-infected patients had elevated kynurenine concentration compared to controls; increased kynurenine levels were associated with altered mental state of HIV patients.
Favre et al. (2010)	HIV+ patients (*n* = 85)	Yes	Plasma, CCS, Tissue, PBMCs	LC-MS/MS	IDO mRNA is upregulated in myeloid dendritic cells from HIV+ patients IDO activity was increased in progressive HIV infection
Byakwaga et al. (2014)	HIV+ patients (*n* = 435)	Yes	Plasma	LC-MS/MS	IDO activity independently predicted poor CD4+ T cell recovery Elevated IDO activity was associated with increased mortality among HIV-infected patients.
Chen et al. (2014)	HIV+ patients (*n* = 76) HIV– patients (*n* = 16)	Yes	Plasma	HPLC-MS	IDO activity significantly declined after treatment; however, it did not reach normal activity.
Page et al. (2014)	HIV+ patients (*n* = 36) Controls (n = 16)	40% on ART	Plasma	HPLC-MS	IDO activity was significantly elevated in HIV-infected patients not receiving antiretroviral therapy compared to patients on treatment.
Jenabian et al. (2015)	HIV+ patients (*n* = 116) Controls (*n* = 12)	Yes	Plasma	HPLC-MS/MS	Early phase of HIV-infection, and/ or patients not receiving ART has high IDO activity. IDO activity declined after initiating treatment. IDO activity was positively associated with CD8+ T cell activation
Bipath et al. (2015)	HIV+ patients (*n* = 105) HIV– patients (*n* = 60)	78% on ART	Plasma	GC-MS	Tryptophan depletion directly correlated with the severity of immune deficiency.
Gaardbo et al. (2015)	HIV+ patients (*n* = 41)	Yes	Plasma	LC-MS/MS	IDO activity decreased after antiretroviral therapy initiation. High IDO activity was associated with increased of Tregs, low naïve T cells, low CD4/CD8 ratio.
Gelpi et al. (2017)	HIV+ patients (*n* = 100) HIV– patients (*n* = 16)	Yes	Plasma	LC-MS/MS	Early initiation of antiretroviral therapy has a beneficial effect on kynurenine/tryptophan ratio set point, and this is associated with reduced immune stimulation.
Chen et al. (2018)	HIV+ patients (*n* = 127) HV– patients (*n* = 25)	Yes	Whole blood	UPLC-MS	HIV viral load positively correlated with IDO activity, immune activation and T cell exhaustion.

*Human Immunodeficiency virus (HIV), Indoleamine 2, 3-dioxygenase (IDO), Positive (+), Negative (–), High-performance liquid chromatography-mass spectrometry (HPLC-MS/MS), Ultra-performance liquid chromatography-mass spectrometry (UPLC-MS/MS), Gas chromatography-mass spectrometry, Real-time polymerase chain reaction (RT-PCR), Cell culture supernatant (CCS), Cerebrospinal fluid (CSF), Antiretroviral therapy (ART), regulatory T cells (Tregs)*.

## IDO Activity in Human Tuberculosis Disease

Despite extensive work in murine and macaque models of TB, only a few studies have investigated IDO-mediated tryptophan catabolism and its metabolites in human TB (Table 2). M. TB has been shown to induce IDO activity *in vitro* (Blumenthal et al., 2012) and *in vivo* (Gautam et al., 2018). Almeida et al. (2009) studied 20 immunological mediators by reverse transcriptase-polymerase chain reaction (rt-PCR) in induced sputa of patients with active TB disease, patients with other lung diseases and controls. The authors found significantly elevated IDO expression in TB patients. The IDO levels decreased more than 500-fold within 2 weeks of beginning of TB treatment. Their work suggested that IDO might be a useful tool to diagnose active disease and monitor the efficacy of TB treatment; however, their studies did not include HIV-infected patients (Almeida et al., 2009). Weiner et al. explored more than 400 metabolites from the serum of latently infected and active TB patients and found that IDO activity was elevated in active TB patients (Weiner 3rd et al., 2012). Li et al. found increased IDO expression and activity in pleural fluid from TB patients (Li et al., 2011). We and others have shown that IDO activity is elevated at the time of TB diagnosis and declines after TB treatment (Almeida et al., 2009; Suzuki et al., 2012, 2013; Adu-Gyamfi et al., 2017) (Table 2). Both HIV-infected and uninfected patients with active TB had elevated IDO activity compared with latent TB infection (Almeida et al., 2009; Suzuki et al., 2012; Adu-Gyamfi et al., 2017; Chen et al., 2018). Additionally, IDO activity showed potential to predict the onset of active disease ahead of major TB symptoms (Adu-Gyamfi et al., 2017) and could be useful in early diagnosis of multi-drug resistance TB (Shi et al., 2019).

**Table 2:** Studies reporting IDO activity or either kynurenine or tryptophan in human active TB disease.

**References**	**Participant demographics**	**Sample type**	**Method of IDO** **quantification**	**Major conclusion (s)**
Almeida et al. (2009)	TB cases (*n* = 30) HIV+, active TB disease (*n* = 4) Other lungs diseases (No-TB, HIV– *n* = 11), Healthcare workers (*n* = 16)	Induced Sputum	RT-PCR	Active TB patients expressed significantly higher IDO compared with patients with other lung disease and health care workers. IDO expression declined over 500-fold change 15 days post-treatment with anti-TB medication.
Li et al. (2011)	TB cases (*n* = 31) Controls (*n* = 35)	Pleural effusion	Cell culture	Inhibiting IDO reversed cytokine production and restored T cell functions *in vitro*.
Weiner 3rd et al. (2012)	Active TB cases (*n* = 44) LTBI (TST+) (*n* = 46) No-TB (TST-) (*n* = 46)	Blood	UPLC/GC–MS/MS Cell culture	Metabolic profiles showed IDO activity increased in TB pathogenesis.
Suzuki et al. (2012)	TB case (n = 174) Controls (*n* = 85)	Serum	LC-MS/MS	Patients with pulmonary TB has elevated serum IDO activity compared to controls. Higher IDO activity independently predicted death of TB patients.
Suzuki et al. (2013)	TB cases (*n* = 92) TB pleurisy (*n* = 34), Malignant pleuritis (*n* = 36) Parapneumonic effusion (*n* = 15)	Pleural effusion	LC-MS/MS	IDO activity may be strongly involved in the pathogenesis of TB pleurisy. IDO activity holds diagnostic significance in TB pleurisy.
Feng et al. (2015)	TB cases (*n* = 120) Non-TB (*n* = 146) Healthy controls (*n* = 105)	Blood	UPLC-MS	Kynurenine and quinolinic acid, which are products of IDO-mediated tryptophan metabolism were among 20 metabolic profiles that indicated active TB disease.
Adu-Gyamfi et al. (2017)	TB-HIV cases (*n* = 32) Controls (HIV+, No-TB) (*n* = 70) Pneumonia (HIV positive) (*n* = 37)	Plasma	UPLC-MS/MS	IDO activity is a plausible diagnostic active TB in HIV infected patients. IDO activity predicted active TB disease months ahead of major TB symptoms, IDO activity declined in all patients after standard TB treatment.
Van Laarhoven et al. (2018)	Discovery cohort: TBM, HIV– patients (*n* = 33) Controls (*n* = 22) Validation cohort: TBM patients (*n* = 101) Controls (HIV-, no TBM) (*n* = 17)	CSF and Serum	UPLC-MS/MS	Increased tryptophan metabolites were found among TB meningitis patients, and it correlated strongly with mortality.
Shi et al. (2019)	Drug susceptible TB cases (*n* = 16) MDR-TB cases (*n* = 16), Controls (No-TB *n* = 11, Lung cancer *n* = 6) No patient or control had HIV infection.	Plasma	LC-MS/MS	Plasma IDO activity could distinguish multi-drug resistance—TB (MDR-TB) from drug-susceptible TB and lung cancer. Higher plasma IDO activity can indicate a higher risk of MDR-TB.

*Tuberculosis (TB), Tuberculous meningitis (TBM), Human Immunodeficiency virus (HIV), Indoleamine 2,3-dioxygenase (IDO), latent Tuberculosis infection (LTBI), Positive (+), Negative (–), High-performance liquid chromatography-mass spectrometry (HPLC-MS/MS), Ultra-performance liquid chromatography-mass spectrometry (UPLC-MS/MS), Gas chromatography-mass spectrometry, Real-time polymerase chain reaction (RT-PCR)*.

## Laboratory Measurement of IDO Activity

IDO can be studied quantitatively by measuring mRNA expression or protein levels or enzymatic activity (product-to-substrate ratio; Li et al., 2011; Mehra et al., 2012; Suzuki et al., 2012, 2013; Huang et al., 2013; Bipath et al., 2015; Zhang et al., 2019). IDO activity can be detected in host fluids including blood, sputum, pleural fluid, cerebrospinal fluid and urine (Almeida et al., 2009; Li et al., 2011; Suzuki et al., 2012, 2013; Bipath et al., 2015; Adu-Gyamfi et al., 2017; Gautam et al., 2018; Yarbrough et al., 2018). IDO mRNA expression has been studied using rt-PCR, and IDO protein has been measured by western blot, radioimmunoassay and immunofluorescence methods (Almeida et al., 2009; Soliman et al., 2013; Zhang et al., 2019). Recently, intracellular staining methods have also been described (Sørensen et al., 2011). For purposes of clinical diagnosis, the current gold standard method for measuring IDO activity is using liquid chromatography-mass spectrometry (Suzuki et al., 2012; Huang et al., 2013; Adu-Gyamfi et al., 2017). Even though the chromatographic-mass spectrometry method has been reliable, it requires substantial laboratory infrastructure and high-grade expertise; hence the facility is not available in frontline diagnostics, especially in resource-constrained, high burden countries (Tiwari et al., 2007). Newer, higher throughput, lower-cost method–enzyme-linked immunosorbent assay (ELISA) are available (Ziklo et al., 2018). The quantitative ELISA method can be performed using cell culture supernatant, serum or plasma. We recently evaluated the ELISA method in comparison with the mass spectrometry method for measuring IDO activity. The ELISA showed good reproducibility and agreement, and could replace mass spectrometry as the analytical method for clinical use. ELISA has an added advantage of small sample volume and the potential of being fully automated compared with mass spectrometry. IDO detection in plasma has shown good discrimination between patients with active compared to latent TB, and may reflect a cumulative picture of intracellular IDO activity over time (Adu-Gyamfi et al., 2017).

## IDO Activity as a Novel Active TB Biomarker in HIV-infection

A biomarker is defined as any characteristic that can be objectively measured as an indicator of health or disease process or as a response to a therapeutic intervention (Group et al., 2001; Phillips et al., 2010; Mcnerney et al., 2012; Walzl et al., 2018). In an infectious disease, biomarkers can be host or pathogen-derived parameters (Phillips et al., 2010; Mcnerney et al., 2012; Wallis et al., 2013). In TB, the lack of a biomarker indicating risk of progression to active disease, particularly in individuals with compromised immunity, presents a massive gap in consolidating efforts to eliminate the disease. Similarly, the absence of a diagnostic TB biomarker or a marker to monitor response to anti-TB therapy cripples the efficiency of global TB control strategies. Despite increased research aimed at finding a reliable, inexpensive and easily quantitated TB biomarker, our lack of full understanding of factors mediating the switch of latent TB to active TB disease is partly to be blamed for lack of an improved TB vaccine and new therapeutics.

Most TB studies have relied on animal models or *ex vivo* M. TB challenge in cells or tissue from active or latently infected patients to understand TB pathology. Some studies have examined metabolic profiles or genes from active TB patients to identify metabolites or genes that are switched on or off, which may be implicated in the transition from latency to active disease in both HIV-infected and uninfected individuals (Weiner 3rd et al., 2012; Scriba et al., 2017; Suliman et al., 2018). Despite dynamic efforts in the search for a sensitive, specific, and reliable TB biomarker, there has been insufficient attention paid to the host pathway of tryptophan metabolism, which is a central player in both HIV infection and TB disease. M. TB, an organism able to synthesize tryptophan, thrives during tryptophan depletion in its microenvironment, while proliferating T cells die *in vitro* (Zhang et al., 2013; Berney and Berney-Meyer, 2017; Gautam et al., 2018). The high level of interferon-γ during TB infection activates IDO-mediated tryptophan catabolism in macrophages (Giacomini et al., 2001; Khan et al., 2016). This phenomenon may result in M. TB's ability to hijack IDO's immunosuppressive actions on the host immune system to cause pathology.

It remains to be determined whether elevated IDO activity is causative of, or only associated with, progressive HIV and TB disease. Some researchers postulate that human co-existence with latent TB infection may be a human evolutionary adaptation to increase host tryptophan in times of low dietary tryptophan availability (Williams and Dunbar, 2013; Williams and Hill, 2017). Intriguingly, M. TB can produce nicotinamide from tryptophan (Fricker et al., 2018). An older laboratory test for distinguishing M. TB from non-tuberculous mycobacteria was known as the niacin test (Ogawa et al., 1968). Isoniazid (INH), one of the most effective anti-TB drugs was developed as a nicotinamide analog for the treatment of TB (Kushner et al., 1952; Murray, 2003a, Mckenzie et al., 1948). Serendipitously, nicotinamide was discovered to be anti-tuberculous even though the mechanism of action remained unknown (Mckenzie et al., 1948; Kushner et al., 1952; Murray, 2003a). Accordingly, these pieces of evidence support the fact that elevated flux through the tryptophan-nicotinamide pathway results in M. TB inhibition.

Currently, several clinical trials are exploring blocking IDO activity for therapeutic use. During infection with *Chlamydia trachomatis*, an intra-macrophage pathogen, which can synthesize tryptophan, blocking IDO activity with levo-1-methyl-tryptophan led to improved susceptibility to doxycycline (Ibana et al., 2011). In TB, mechanistic studies have demonstrated that blocking IDO activity in Simian immunodeficiency virus (SIV) infection improved T cell number and functions (Boasso and Shearer, 2007; Boasso et al., 2009). Blocking IDO activity in combination with antiviral therapy significantly reduced the SIV viral load in plasma and lymph nodes of treated animals (Boasso et al., 2009). Recently, Gautam et al. (2018) demonstrated *in vivo* that M. TB induces IDO expression in the lungs of macaques and mice with active TB disease. In the macaque, inhibition of IDO activity led to a reduced M. TB burden, pathology and improved animal survival (Gautam et al., 2018). Together, these results suggest that there exists a potential for using IDO inhibitors as an effective and host-driven therapy in TB. IDO inhibition as a therapeutic strategy against various cancers has yielded encouraging results (O'brien et al., 2003; Muller et al., 2005; Hanafi et al., 2014; Spranger et al., 2014; Austin and Rendina, 2015; Prendergast et al., 2017, 2018). IDO inhibitors are thus readily available and include some agents already licensed by the US Food and Drug Administration (Jeong et al., 2013).

In summary, IDO-mediated tryptophan catabolism via the kynurenine-nicotinamide pathway represents a common axis upregulated in HIV infection and TB disease. Rather than being uniquely elevated in TB, cumulative elevation of IDO in HIV and TB may explain the extraordinarily high rate of progression to active TB in HIV-infected patients. It is unclear whether elevated IDO is causative of progression to active disease or a compensatory response to the microbe. There is much promise in using metabolites from the IDO-mediated tryptophan pathway to diagnose active TB and monitor anti-TB treatment. IDO holds promise as a TB biomarker in both HIV-positive and negative patients, but further confirmatory studies are urgently needed. Regarding new TB drug targets, it remains speculative whether IDO inhibition would be beneficial. Further exploration of this pathway is necessary to better understand TB pathogenesis and discover novel host biomarkers and potential drug targets for either TB or HIV disease.

## Author Contributions

All authors listed have made a substantial, direct and intellectual contribution to the work, and approved it for publication.

### Conflict of Interest

The authors declare that the research was conducted in the absence of any commercial or financial relationships that could be construed as a potential conflict of interest.
